# Variations in the metabolome in response to disease activity of rheumatoid arthritis

**DOI:** 10.1186/s12891-016-1214-5

**Published:** 2016-08-22

**Authors:** Zuzana Tatar, Carole Migne, Melanie Petera, Philippe Gaudin, Thierry Lequerre, Hubert Marotte, Jacques Tebib, Estelle Pujos Guillot, Martin Soubrier

**Affiliations:** 1 Rheumatology Department, CHU Gabriel Montpied, 58 rue Montalembert B.P. 392, 63011 Clermont-Ferrand, France; 2 Metabolomics Platform, INRA, Clermont-Ferrand, France; 3 Rheumatology Department, CHU Grenoble–Hôpital Sud, Echirolles, France; 4 CHU Bois-Guillaume, Rouen, France; 5 INSERM 1059, Université de Lyon, Saint-Etienne, France; 6 Rheumatology Department, CHU Saint-Etienne, Saint-Etienne, France; 7 Rheumatology Department, Centre Hospitalier Lyon-Sud, Pierre-Bénite, France

**Keywords:** Rheumatoid arthritis, Metabolomics, Anti-TNF-alpha therapy, Drug response biomarkers

## Abstract

**Background:**

Anti-Tumor Necrosis Factor (TNF) therapies are able to control rheumatoid arthritis (RA) disease activity and limit structural damage. Yet no predictive factor of response to anti-TNF has been identified. Metabolomic profile is known to vary in response to different inflammatory rheumatisms so determining it could substantially improve diagnosis and, consequently, prognosis.

The aim of this study was to use mass spectrometry to determine whether there is variation in the metabolome in patients treated with anti-TNF and whether any particular metabolomic profile can serve as a predictor of therapeutic response.

**Methods:**

Blood samples were analyzed in 140 patients with active RA before initiation of anti-TNF treatment and after 6 months of Anti-TNF treatment (100 good responders and 40 non-responders). Plasma was deproteinized, extracted and analyzed by reverse-phase chromatography–QToF mass spectrometry. Extracted and normalized ions were tested by univariate and ANOVA analysis followed by partial least-squares regression-discriminant analysis (PLS-DA). Orthogonal Signal Correction (OSC) was also used to filter data from unwanted non-related effects. Disease activity scores (DAS 28) obtained at 6 months were correlated with metabolome variation findings to identify a metabolite that is predictive of therapeutic response to anti-TNF.

**Results:**

After 6 months of anti-TNF therapy, 100 patients rated as good responders and 40 patients as non-responders according to EULAR criteria. Metabolomic investigations suggested two different metabolic fingerprints splitting the good-responders group and the non-responders group, without differences in anti-TNF therapies. Univariate analysis revealed 24 significant ions in positive mode (*p* < 0.05) and 31 significant ions in negative mode (*p* < 0.05). Once intersected with PLS results, only 35 ions remained. Carbohydrate derivates emerged as strong candidate determinants of therapeutic response.

**Conclusions:**

This is the first study describing metabolic profiling in response to anti-TNF treatments using plasma samples. The study highlighted two different metabolic profiles splitting good responders from non-responders.

## Background

Anti-Tumor Necrosis Factor (TNF) drug therapies were first introduced in rheumatoid arthritis (RA) treatment more than 10 years ago. Anti-TNF agents are able to control RA disease activity and limit structural damage. However, a third of patients do not respond to treatment, prompting efforts to find predictive factors of therapeutic response in order to optimize patient management and to limit the systematic use of these drugs. Since the combination of potential side effects like infections, reactivation of tuberculosis and basocellular carcinomas, with high cost (averaging €12000 per year per patient) can often outweigh the therapeutic benefits. Despite this effort, no predictive factor of response to anti-TNF has yet been clearly identified [1–3].

The metabolome, i.e. the complete set of metabolites, varies in response to different diseases, and so can potentially make diagnosis and treatment easier. Metabolomic profile is known to vary in response to different inflammatory rheumatisms, so determining it could substantially improve diagnosis and, consequently, prognosis [4]. In patients with inflammatory rheumatism, studying the metabolome could facilitate earlier diagnosis of rheumatoid arthritis (RA) and subsequently better management and prognosis.

The aim of this study was to determine whether there is variation in the metabolome in patients treated with anti-TNF and whether any particular metabolomic profile can serve as a predictor of therapeutic response.

## Methods

### Study population

All RA patients were aged >18 years and met the 1987 revised classification criteria of the American College of Rheumatology [5]. All patients has failed to respond to treatment with at least one disease-modifying antirheumatic drug, and were treated with methotrexate at a dose of at least 7.5 mg weekly. Prednisolone was allowed provided the dose remained stable and did not exceed 10 mg daily.

Patients with RA requiring anti-TNF-alpha treatment were recruited in the Rheumatology department of university teaching hospitals at Clermont-Ferrand, St-Etienne, Lyon, Rouen, and Grenoble, all in France. A plasma bank was created from samples taken from every patient. Anticitrullinated protein antibodies (ACPA) were detected with enzyme-linked immunoabsorbent assay (ELISA) and were considered positive at a serum concentration ≥5 IU/ml. Rheumatoid factor (RF) was measured by the particle-enhanced immunonephelometry with the lower level of detection of 10 IU/ml.

Here we chose to consider only good-responders and non-responders in order to increase the chances of finding significant differences in metabolic profiles. Indeed, only baseline blood samples (collected before the initiation of anti-TNF treatment : “M0”) of good-responders and non-responders were used for the metabolomics analysis. In total, 140 venous plasma samples were withdrawn, in a non-fasting state, before the initiation of anti-TNF-alpha treatment (M0) and frozen at −80 °C until analysis.

RA patients received anti-TNF therapy (infliximab, abatacept or etanercept) and this treatment was kept unchanged for at least the first 6 months. Initially, all the patients had active RA at M0 (DAS-28 >3.2) requiring biological therapy and underwent disease severity assessments (DAS-28 ESR, DAS-28 CRP, Health Assessment Questionnaire scores [6]). Anti-TNF was started at usual doses and by standard administration. Three different anti-TNF therapies were used : infliximab, adalimumab or etanercept. Six months after initiation of treatment (M6), DAS28 was assessed to classify therapeutic response according to EULAR criteria [7] and thus create three groups: good responders, moderate responders, and non-responders. A good clinical response in RA was defined as a DAS28 ≤3.2 and a >1.2-point improvement in DAS28 after anti-TNF therapy [8]. A non-response in RA was defined as a DAS28 ≥5.1 and a <0.6-point improvement in DAS28 after the anti-TNF-alpha therapy [8].

Disease activity scores obtained at M6, and metabolome variation findings were cross-correlated to identify a metabolomics fingerprint that is predictive of therapeutic response to anti-TNF-alpha therapy.

The study was approved by the appropriate institutional review boards/independent ethics committees (*Committee of Patient*’*s Protection Grenoble 05-CHUG3 et Sud-Est 6 –AU731*), and was carried out in accordance with the ethical principles of the Declaration of Helsinki. All patients gave written, informed consent.

### Metabolomics analysis

The blood samples were first deproteinized and extracted, then analyzed by reverse-phase liquid chromotography–electrospray QToF mass spectrometry. The metabolic profiles were collected using positive and negative ionization, in full-scan mode, on a mass range from 90 to 1000 m/z. The data were then centroided and corrected before reprocessing to obtain a matrix containing retention times, exact masses and intensities of potential markers.

A preliminary quality analysis of the data performed by principal component analysis detected the possible effect of fouling of the ionization source of the mass spectrometer during the sample injection series. These effects were checked and corrected using quality control samples corresponding to the pools of all the analyzed samples. After linear regression modeling of this instrumental drift on quality control samples, the model thus obtained was used to correct all extracted ion intensities.

The list of extracted and normalized ions was considered in univariate analysis. The results from metabolomic investigations were analyzed by ANOVA and cross-compared against partial least-squares regression-discriminant analysis data (PLS-DA). Orthogonal Signal Correction (OSC) was also used to filter data from unwanted non-related effects. Results were considered statistically significant if *p* < 0.05 and if VIP (Variable Importance of the Projection) >1.5. Ions were identified by annotation in the HMDB database.

### Statistical analysis

This was an exploratory and multicentric study. Biostatistical analyses followed the design of a case-control study with binary analysis (“case” subjects are good-responders according to EULAR response criteria; “control” subjects are non-responders according to EULAR response criteria).

The justification for the number of subjects was based primarily on ability to recruit, sample sizes of princeps publications, and the experience of the services involved in this research. In order to identify predictive metabolites of therapeutic response, it was necessary to include 120 patients (for a bilateral risk of error α equal to 5 % and a power ≥90 %) considering:

1. an in-population response rate of 60 %: distribution was 70 % responders (breaking down as 35 % very good responders and 35 moderate responders) and 30 % non-responders;
2. the rules for determining the required number of subjects involved in the study of predictors of a binary response variable as defined by Harrell et al. [9].

Concerning power estimation for each metabolite, we explored different simulations, as proposed by Cohen [10], using as starting point the conventional effect sizes (ES) for the Student test: small (ES = 0.2), mean (ES = 0.5) and high (ES = 0.8). Given the sample size of this study (i.e. 70 cases and 50 controls), the expected ES was 0.6 for α = 5 % and β = 10 %.

Statistical analyses were performed in STATA v11 (STATA Corp, College Station, TX). Numerical outcomes were expressed as mean ± SD (for Gaussian distributions). Nominal outcomes were expressed as raw values and percentages. Level of significance was set at 5 %.

## Results

After 6 months of anti-TNF therapy, 100 patients were considered good-responders and 40 patients as non-responders according to EULAR criteria [7]. The baseline characteristics of the patients are summarized in the Table 1. There was no difference in response to anti_TNF treatment between patients with or without rheumatoid factors or anti-citrullinated peptide.

**Table 1:** Baseline characteristics of rheumatoid arthritis patients by response to anti-TNF therapy at 6 months

	Good responders (*n* = 100)	Non-responders (*n* = 40)	*P*-value
Age, years (mean ± SD)	50 ± 14	57 ± 13	0.44
Female (%)	76 (76)	31 (77.5)	1.00
Disease duration (months, range)	9 (3.3–15.3)	10 (3.7–12.8)	0.54
Infliximab (%)	13 (13)	7 (17)	0.64
Adalimumab (%)	29 (29)	17 (43)	0.32
Etanercept (%)	58 (58)	16 (40)	0.82
DAS-28 M0 (Mean ± SD)	4.8 ± 0.9	5.0 ± 1.2	-
DAS-28 M6 (Mean ± SD)	2.2 ± 0.6	4.8 ± 1.2	-
RF positivity (%)	77.4	70	0.25
ACPA positivity (%)	75	62	0.18
ERS (mm/h)	32 ± 25	35 ± 23	0.37
CRP (mg/l)	17 ± 19	21 ± 18	0.45
Bone erosion (%)	38	31	0.37

*RF* rheumatoid factor, *ACPA* anticitrullinated protein antibodies, *ERS* erythrocyte sedimentation rate, *CRP* C-reactive protein

**Fig. 1:**
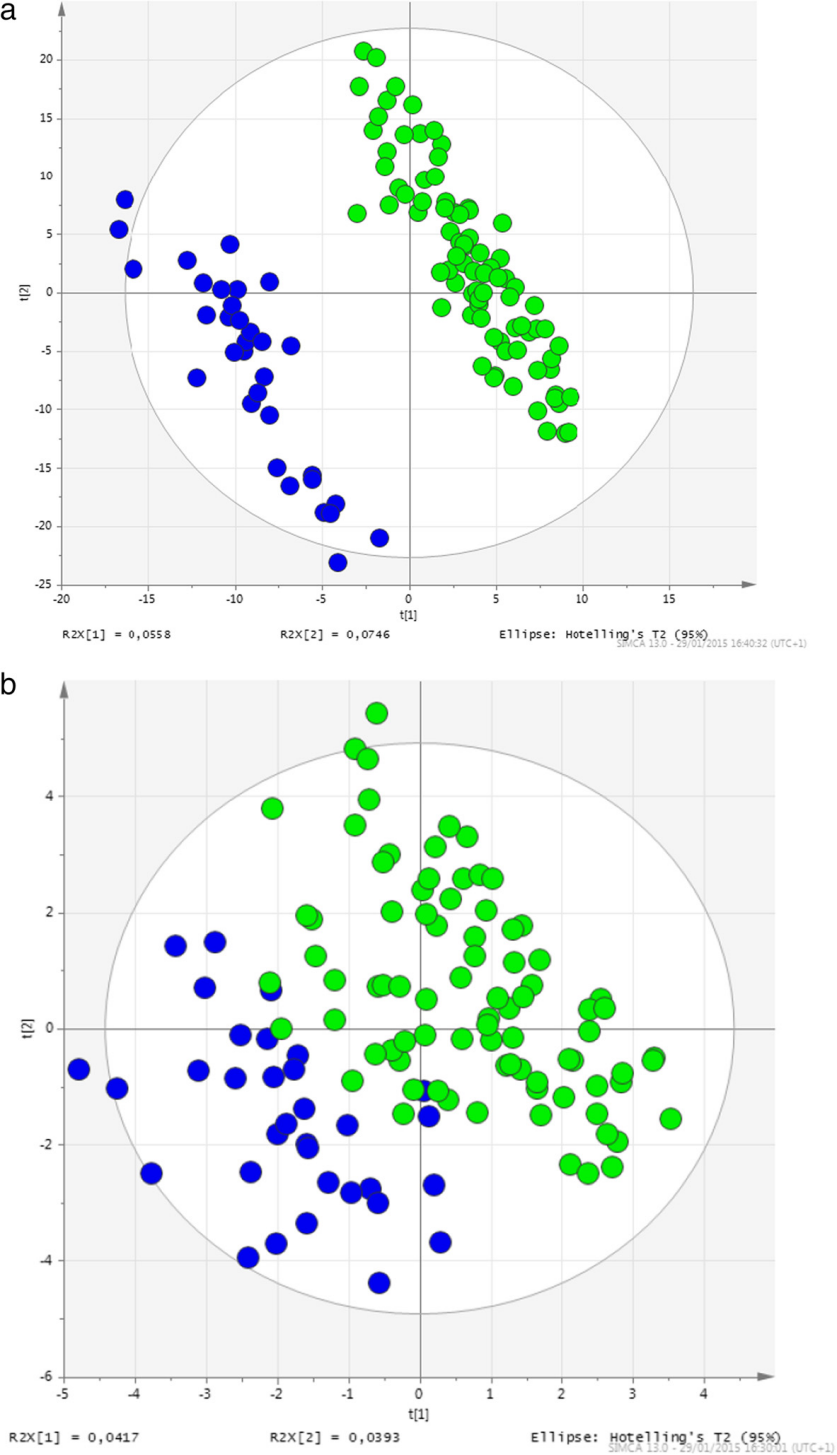
Metabolomic fingerprinting distinguished between baseline plasma samples from RA patients demonstrating good response to anti-TNF agents (*green circles*) and no response (*blue circles*) at 6 months in positive (**a**) and negative (**b**) mode analysis

Metabolomic investigations suggested two different metabolic fingerprints segregating good-responders group from non-responders group (Fig. 1). There is a concrete global effect of discriminative ions, even though we were unable to report any single discriminating biomarker that could simplify routine management of RA.

The univariate analyses revealed 24 significant ions in positive mode (*p* < 0.05) and 31 significant ions in negative mode (*p* < 0.05). Once intersected with PLS results, only 35 ions remained. The ions-of-interest were then identified by querying the HMDB database (Table 2). Carbohydrate derivates (D-glucose, D-fructose, sucrose, and maltose) emerged as determinants of therapeutic response. Because of lack of pertinent biomarkers, no absolute quantifications and identifications of different metabolites were proceeded.

**Table 2:** Baseline plasma metabolites most strongly correlated with response to TNF antagonists using partial least-squares regression and ANOVA (VIP >1 with *p* < 0.1 and VIP >1.5 with *p* < 0.05)

Ion mass (Da)	RT (min)	Formula
Positive-mode analysis		
181.070	5.2	C6H12O6, C42H44FeN8O8S2R4, C6H11O6R, CH2NOR, NH2R, C10H17O10PR2, C5H9O5R, C38H37FeN6O6SR2, C7H8N4O2,
224.129	4.2	C12H17NO3
268.148	12.7	C16H17N3O
460.293	6.0	C29H37N3O2
481.350	13.2	C28H48O6
482.359	13.3	C24H52NO6P
583.257	9.9	C33H34N4O6
Negative-mode analysis		
115.038	4.6	C5H8O3
128.033	1.4	C5H7NO3, C5H12O12P3R
181.054	5.5	C9H10O4
203.022	0.9	C7H8O7, C6H10NO3SR, C12H9ClO
221.156	12.9	C14H22O2
249.115	10.3	C14H18O4
281.249	16.3	C18H34O2
285.183	7.8	C19H26O2, C14H26N2O4, C19H37N5O7
313.067	1.2	C17H14O6
335.223	11.3	C20H32O4, C10H18O13P2R2, C20H34NO15R, C6H13NO7PR, C7H13NO9PR, C10H15NO11PR3, C4H7O7PR2,
341.111	1.3	C12H22O11, C24H39N7O18P3SR, C27H40N10O23P4R, C10H14N5O8P, C17H24N5O10P, C8H6NO3R, NH2R
425.289	6.7	C20H38N6O4
466.296	12.0	C29H41NO4
87.009	1.3	C3H4O3

*RT* retention time, *VIP* variable importance in the projection

Despite of the discriminatory power of ions, the construction of a PLS model based only on the most significant ions did not allow sufficient discrimination: only a global effect could be linked to the therapeutic response.

There was no difference between different groups of anti-TNF therapies (infliximab, etanercept and adalimumab. We found no interaction of the metabolome with age, sexe and rheumatoid factors or anti-citrullinated peptide status, use of steroids or methotrexate. We have no sufficient data about smoking status, glucose blood levels or lipidic profile.

## Discussion

This is the first study to describe metabolomic profiling in response to anti-TNF treatments using plasma samples. Our study highlighted two different metabolomic profiles splitting the good-responders group from the non-responders group. There is a concrete global effect of discriminative ions.

The major limit of our study is the impossibilty to identify any particular biomarker. However, this is an original study and despite our originality we could not found discriminant biomarkers in our patient’s sample. To our knowledge, there is no other study published with more significant results.

Choice of sample is a fundamental and potentially decisive factor: indeed, analyses of blood or plasma can prove more complicated than metabolomic investigations with urine samples as they contain all the metabolites from different whole-body pathways. Opting for urine analysis could simplify metabolomic measurement and results interpretation in further research [11]. Nevertheless, metabolomic fingerprinting offers new prospects for finding predictive biomarkers of response to biological agents.

An other limit of our study is the absence of negative controls. Indeed, metabolomic approach limits the number of different samples that can be analysed at the same time and our objective was to find metabolomic differences between good and no responders. We choose to analyse more patients than negative controls in order to show more powerful results.

Contrary to other publications, the main metabolomic differences between responder and non-responder groups concerned carbohydrate derivatives. Nevertheless, recent advances in glycomics and glycol biomarker profiling show direct or indirect associations between glycosylation modifications and autoimmune disturbances in RA: peptide epitope/glycol epitope cross-reactivity, neo-expression of normally-restricted glycans, sugar induction of inappropriate processing and presentation of self-antigens to T-lymphocytes and conformational glycomodification leading to unmasking of antigenic epitopes [12].

Indeed, immune response can be strongly modulated by induced changes in glycosylation site (galactosylation, sialylation or fucosylation) in the constant domain of the IgG Fc region [13]. Animal and human studies suggest that aberrant glycosylation of IgG plays a key role in RA pathophysiology [14–16]. For example, increased fucosylation despite low galactose levels of the IgG Fc region strongly modifies antibody binding capacities and induces abnormal inflammatory reaction [14, 17]. Moreover, increased rate of glycosylated IgG was correlated with 10-year structural prognosis of RA diagnosis with 95 % specificity and 90 % sensitivity when associated with rheumatoid factor [14]. Glycomodifications have even distinguished early RA from other rheumatic diseases [16]. Also, Newkirk et al. reported that the rheumatoid factor avidity was significantly correlated with the presence of the glycoform of IgG lacking galactose in both circulating and immune complexes-derived IgG [18].

Glyco-biomarkers also reflect RA activity and prognosis as they are correlated with rheumatoid factor, tender joint score, and frequency of subcutaneous nodules as well as structural damage [19–22]. Moreover, levels of glycosylated IgG decreased and became normalized with anti-TNF treatment [23] or with acquired remission [24]. These results are especially consistent and coherent with our findings.

Finding predictive factors of response to biological therapy is central to the management of RA patients, both in the short term to enable rapid relief of pain and in the long term as it is now established that persistence of RA disease activity is correlated with structural damage that can lower the functional prognosis [25–27]. The potential value of the metabolome in the diagnosis of chronic inflammatory rheumatic diseases has been investigated in animal models and in human subjects. Analysis in the RA murine model (*K/BxN transgenic mouse)* [28] found that the specific metabolomic profile (nucleic acids, amino acids, reactive derivatives of oxygen, fatty acids, and the enzymes involved in lipolysis and methylation) was significantly different from that in control mice (*p* = 0.00075), and evidenced 18 metabolites out of the 59 initially studied.

In humans, Madsen et al. [29] showed the value of the metabolome in the early diagnosis of RA established even before expression of the anti-CCP antibodies. Here, mass spectrometry analysis of the metabolome established RA diagnosis with a sensitivity of 93 % and a specificity of 70 %. The metabolomic profile in RA patients compared to healthy controls showed an increase in certain biomarkers (3-phospho-glyceric acid, d-ribofuranose and hypoxanthine) and a decrease in others (histidine, threonic acid, threonine, methionine, cholesterol and asparagine).

The metabolome appears to vary in response to RA disease activity. Lauridsen et al. [30] studied the serum metabolome by NMR mass spectrometry in 47 RA patients (23 with active RA and 24 with RA in remission) and 51 control subjects for one year, and reported a significant difference (*p* = 0.0007) in metabolomic profile between patients with active RA and those in remission. They identified several potential markers of RA disease severity, including total cholesterol, lactates, acetyl glycoprotein and lipid derivatives. The difference between the two groups was non-significant (*p* = 0.91) at 31 days after initiation of effective treatment in patients with active RA. The metabolome of the two patient groups remained different from that of the control group.

Nevertheless, studying the metabolome could not only improve the diagnosis of a disease but also predict the tolerance and effectiveness of certain treatments [31]. For example, analysis of the urinary metabolome was able to predict the digestive toxicity of non-steroidal inflammatory drugs in rats [32].

However, there is only one documented report on urine metabolomic variations in RA patients on anti-TNF-alpha treatment. Kapoor et al. [11] showed that variations in metabolomic profile correlated with response to anti-TNF agents according to EULAR criteria with a good sensitivity (88.9 %) and specificity (85.7 %). Variation of expression of histamine, glutamine, xanthurenic acid and ethanolamine were particularly significant and predictive. There is no published metabolomic analysis of plasma samples concerning response to anti-TNF-alpha treatment in RA patients.

## Conclusion

This is the first study describing metabolic profiling in response to anti-TNF treatments using plasma samples. The study highlighted two different metabolic profiles splitting good responders from non-responders. Our metabolomic approach needs to be completed by screening larger cohorts of patients and investigating outcomes with other biologic agents in patients with severe RA. Metabolomic analysis remains expensif but in case of identification of pertinent biomakers, classic quantitative analysis could be used in clinical practice.

## Abbreviations

ANOVA: Analysis of variance
Anti-CCP: Anti-cyclic citrullinated peptide
CRP: C-reactive protein
DAS 28: Disease activity scores
ESR: Erythrocyte sedimentation rate
EULAR: European League Against Rheumatism
HMDB: Human metabolome database
IgG: Immunoglobulin G
NMR: Nuclear Magnetic Resonance
OSC: Orthogonal Signal Correction
PLS-DA: Partial least-squares regression-discriminant analysis
QTOF: Quadrupole-time-of-flight
RA: Rheumatoid arthritis
RT: Retention time
SD: Standard deviation
TNF: Tumor Necrosis Factor
VIP: Variable importance in the projection
